# Heterogeneity and frequency of BRAF mutations in primary melanoma: Comparison between molecular methods and immunohistochemistry

**DOI:** 10.18632/oncotarget.14094

**Published:** 2016-12-21

**Authors:** William Bruno, Claudia Martinuzzi, Virginia Andreotti, Lorenza Pastorino, Francesco Spagnolo, Bruna Dalmasso, Francesco Cabiddu, Marina Gualco, Alberto Ballestrero, Giovanna Bianchi-Scarrà, Paola Queirolo, Federica Grillo, Luca Mastracci, Paola Ghiorzo, on behalf of the Italian Melanoma Intergroup (IMI)

**Affiliations:** ^1^ Department of Internal Medicine and Medical Specialties (DiMI), University of Genoa and IRCCS AOU San Martino-IST, Genoa, Italy; ^2^ Department of Medical Oncology, IRCCS AOU San Martino-IST, Genoa, Italy; ^3^ Department of Pathology, IRCCS AOU San Martino-IST, Genoa, Italy; ^4^ Department of Surgical and Diagnostic Sciences, Pathology Unit, University of Genoa and IRCCS AOU San Martino-IST, Genoa, Italy

**Keywords:** BRAF, primary melanoma, PNA-clamping, immunohistochemistry, heterogeneity

## Abstract

Finding the best technique to identify *BRAF* mutations with a high sensitivity and specificity is mandatory for accurate patient selection for target therapy. *BRAF* mutation frequency ranges from 40 to 60% depending on melanoma clinical characteristics and detection technique used.

Intertumoral heterogeneity could lead to misinterpretation of *BRAF* mutational status; this is especially important if testing is performed on primary specimens, when metastatic lesions are unavailable.

Aim of this study was to identify the best combination of methods for detecting *BRAF* mutations (among peptide nucleic acid – PNA-clamping real-time PCR, immunohistochemistry and capillary sequencing) and investigate *BRAF* mutation heterogeneity in a series of 100 primary melanomas and a subset of 25 matched metastatic samples.

Overall, we obtained a *BRAF* mutation frequency of 62%, based on the combination of at least two techniques. Concordance between mutation status in primary and metastatic tumor was good but not complete (67%), when agreement of at least two techniques were considered. Next generation sequencing was used to quantify the threshold of detected mutant alleles in discordant samples. Combining different methods excludes that the observed heterogeneity is technique-based. We propose an algorithm for *BRAF* mutation testing based on agreement between immunohistochemistry and PNA; a third molecular method could be added in case of discordance of the results. Testing the primary tumor when the metastatic sample is unavailable is a good option if at least two methods of detection are used, however the presence of intertumoral heterogeneity or the occurrence of additional primaries should be carefully considered.

## INTRODUCTION

The discovery of *BRAF* mutations in melanoma and the development of specific *BRAF* inhibitors has led to significant advances in treatment of metastatic melanoma, which has now been further improved by the use of new immunologic therapy [1]. Melanoma patients with metastatic or unresectable disease, according to recent NCCN guidelines [2], should be tested for *BRAF* V600 mutations to evaluate therapy options. However, there is no consensus on the best *BRAF* testing method, except for the need for a certified laboratory. In Italy, national guidelines from the Italian association of medical oncology and Pathology (AIOM-SIAPEC) point to standardization of different testing methods with internal controls, fast turnaround time of results, and the additional value of CE-IVD techniques [3].

*BRAF* mutation rate in melanoma ranges from 40 to 60% of cases [4–7]. This difference, besides reflecting clinical characteristics of the cohort studied, could also be due to the different methods used: a wide range of detection techniques is available, including classical capillary sequencing, pyrosequencing, next generation sequencing techniques (NGS), several real-time PCR (RT-PCR) based methods and immunohistochemistry (IHC) antibodies [8–14]; some of these methods have obtained CE-IVD certification.

Since the approval of *BRAF* inhibitors for the treatment of metastatic melanoma, the importance of *BRAF* heterogeneity in melanoma samples has gained much attention [15]. Tumour heterogeneity and the evolution of branching tumour subclones are a challenge even for multi-targeted therapies and intermittent regimens [16]. Currently, general advice is to test metastatic melanoma for the presence of *BRAF* mutation in the most recent metastatic sample. In some instances, biopsy from metastasis is unavailable, so checking mutational status in the primary tumor has been proposed as an alternative [17]. The consistency in *BRAF* mutation detection among primary and metastatic tumors is however still debated [18–20].

In addition, the clinical significance of finding *BRAF* mutation using a high sensitivity technique in a low number of cells in the primary tumor is still unclear, especially when not confirmed in the metastatic sample. Indeed, a different cell clone, driven by other mutations, could be positively selected and responsible for progression and the development of the metastasis [21].

Currently, data on primary melanoma are scarce, as well as data on matched primary and metastatic melanomas tested with several high sensitivity techniques [21–24]. Generally, IHC staining shows good consistency between primary and metastatic samples, although limited by a lower sensitivity compared to recent molecular techniques, which have, conversely, the disadvantage of relying on DNA quantity and quality [25].

Overall, while recent data applying IHC point to intra and inter tumor homogeneity of *BRAF*-mutated expression, the issue of genetic heterogeneity is still debated [23].

Aim of this study was to identify the best combination of methods for detecting *BRAF* mutations and investigate *BRAF* mutation heterogeneity within 100 primary melanomas and between primary melanoma and a subset of 25 matched metastatic samples. Furthermore, we aim to propose a reliable and efficient scheme for diagnostic mutation testing.

## RESULTS

### Cohort characteristics

Our cohort included 94 primary cutaneous melanomas, 4 mucosal melanomas and 2 uveal melanomas. Median follow-up for survivors was 74 months. During this period, 22 patients relapsed and 19 patients died, of which 13 deaths were melanoma-related. Twelve patients developed multiple melanomas. A summary of patients characteristics is shown in Table 1. In addition 25 metastatic samples, from 15 out of the 22 patients who relapsed during follow-up, were analyzed. Of these, 19 samples were metastatic lymph nodes, and 6 were distant metastases. In 5 patients, lymph nodes were excised at the same time as the primary.

**Table 1:** Clinical and pathological features of the 100 primary melanomas in the study cohort

	*Number of Patients(n=100)*
***Gender and age***	
Male	45
Female	55
Median Age	52 years
***Primary tumor site***	
Trunk	50
Upper limb	16
Lower limb	24
Head and Neck	4
Uveal	2
Mucosal	4
***Breslow thickness***	
<1mm	38
1-2mm	23
2-4mm	17
>4mm	16
Unclassified*	6
Median tumor thickness	1,7
***Ulceration***	
Yes	23
No	77
***Histological subtype***	
SSM	59
Nodular	20
Acral-lentiginous	2
Other	19
***Tumor stage***	
I-II	86
III-IV	14
***Prognostic features***	
Median follow-up	74 months (range 47 Q1 -158 Q3)
Alive	81
Dead	19
Melanoma-related deaths	13
Multiple primary melanomas	12
Relapse	22

* The unclassified cases correspond to 2 uveal and 4 mucosal melanomas, not classified using Breslow. (SSM: superficial spreading melanoma)

### BRAF mutation analysis, using three different techniques, reveals a high percentage of V600 mutations in primary melanomas

**Figure 1:**
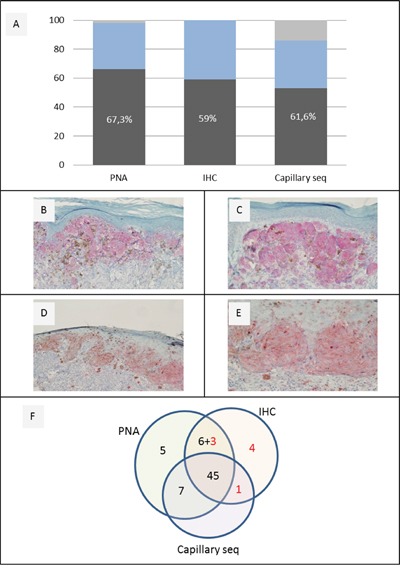
A combination of IHC and molecular techniques reveals a high percentage of V600 mutations **A.** BRAF V600 different mutation rate and yield in 100 primary melanomas tested by PNA, IHC and capillary sequencing. **B-E.** representative IHC analysis of BRAF positive cases [Magnification 20x (B,D); 40x (C,E)]. **F.** Comparison among 100 primary melanomas, which gave a positive successful result with at least one method. Black Numbers indicate BRAF V600 positive cases obtained with each single technique; red numbers show those additional BRAF V600 positive cases obtained with one or two techniques only.

We analyzed 100 FFPE samples from primary melanoma with three different techniques to determine the mutational status of *BRAF* and test which could be the most sensitive method to identify mutations in the *BRAF* V600 codon (Supplementary Table 1 and Figure 1). We chose PNA clamping real-time PCR (here named PNA) and capillary sequencing, to compare the sensitivity of the IHC antibody specific for the *BRAF* V600E mutated protein to molecular techniques.

PNA identified 66 *BRAF* V600 mutated samples out of 98 (67,3%) and 32 wild type (33%); two samples gave invalid results (Figure 1A).

IHC was available for every primary melanoma sample, identifying 59 mutated samples with positive staining (59%) and 41 with negative staining. Among the positive samples, 52 showed an homogeneous staining pattern, while 7 showed an heterogeneous pattern (% of stained cells <95%), in a percentage variable between 10 and 80% (Figure 1A-1E).

Classical capillary sequencing was successful in 86 samples out 100, identifying 53 mutated samples (61,6%); 51 (96%) presented the canonical *BRAF* V600E mutation (c.1799 T>A), one (2%) presented the same amino acidic change V600E due to the less frequent substitution c.1799_1800delTGinsAA, (also called V600E2) and one (2%) the *BRAF* V600K mutation (c.1798_1799delGTinsAA).

Overall, *BRAF* mutation rate in 100 primary melanoma samples was 67,3% when analyzed by PNA, and 59% when the V600E mutation was assessed using the VE1 specific antibody for IHC staining. The classical capillary sequencing technique is sensitive (61,6%) but shows poor performance as 14 cases were not amplified. (Figure 1A). At least one of these highly sensitive methods identified a positive result in 71% of cases.

Sanger sequencing, PNA analysis and IHC provided the same results in 63 cases (46 positive and 21 negative for BRAF mutation). *BRAF* mutation rate was 62% if we consider the results obtained with at least two methods. Overall, agreement among the 3 methods was substantial (Light's k= 0.701, z=3.95, p=7.77^e-05^).

Interestingly, two samples invalid at PNA analysis were found to be positive by IHC (and one of them also at capillary sequencing) (Figure 1F and Supplementary Table 1).

### Characterization of BRAF mutational status in matched primary and metastatic melanomas reveals a good consistency

We analyzed 25 samples of metastatic melanomas, coupled with their primary tumors, deriving from 15 of the 22 patients who relapsed, using the same three methods mentioned above for the primary samples: PNA, IHC and capillary sequencing (Table 2 and Table 3)

**Table 2:** BRAF mutational status in matched primary and metastatic melanomas reveals a good consistency

*Case n*.		*Primary melanoma*	*M1*	*M2*	*M3*	*Intrapatient Concordance*	*Intrapatient Overall Concordance*
**4**	***PNA***	V600	wt			+/−	
	***IHC***	weak	weak			+/+	+/−
	***Capillary seq***	V600E	wt			+/−	
	***Cell %/Localization***	20/Upper limb	70/LN				
**13**	***PNA***	V600	wt	wt		+/−	
	***IHC***	weak	negative	negative		+/−	+/−
	***Capillary seq***	V600E	wt	wt		+/−	
	***Cell %/Localization***	50/Lower limb	50/LN	50/LN			
**18**	***PNA***	V600	V600	V600	n.a.	+/+	
	***IHC***	weak	moderate	strong	moderate	+/+	+/+
	***Capillary seq***	WT	V600E	V600E-	n.a.	-/+	
	***Cell %/Localization***	80/Lower limb	80/Skin	70/Skin	70/LN		
**25**	***PNA***	V600	n.a.	wt	n.a.	+/−	
	***IHC***	moderate	moderate	moderate	moderate	+/+	+/−
	***Capillary seq***	V600E-	wt	wt	n.a.	+/−	
	***Cell %/Localization***	80/Lower limb	70/Skin	40/LN	30/LN		
**39**	***PNA***	V600	V600	V600		+/+	
	***IHC***	weak	moderate	strong		+/+	+/+
	***Capillary seq***	WT	V600E	V600E		-/+	
	***Cell %/Localization***	50/Trunk	80/LN	80/Skin			
**46**	***PNA***	wt	wt			−/−	
	***IHC***	negative	negative			−/−	−/−
	***Capillary seq***	n.a.	wt			n.a.	
	***Cell %/Localization***	80/Trunk	80/LN				
**55**	***PNA***	V600	wt			+/−	
	***IHC***	negative	negative			−/−	+/−
	***Capillary seq***	V600E	wt			+/−	
	***Cell %/Localization***	10/Upper limb	80/LN				
**67**	***PNA***	V600	V600			+/+	
	***IHC***	weak	weak			+/+	+/+
	***Capillary seq***	n.a.	V600K			n.a.	
	***Cell %/Localization***	60/Trunk	80/LN				
**70**	***PNA***	V600	V600			+/+	
	***IHC***	weak	strong			+/+	+/+
	***Capillary seq***	V600E	V600E			+/+	
	***Cell %/Localization***	70/Trunk	40/LN				
**72**	***PNA***	V600	V600	V600		+/+	
	***IHC***	moderate	strong	strong		+/+	+/+
	***Capillary seq***	V600E2	V600E2	V600E2		+/+	
	***Cell %/Localization***	80/Trunk	60/LN	60/LN			
**75**	***PNA***	V600	wt	wt		+/−	
	***IHC***	negative	negative	negative		−/−	+/−
	***Capillary seq***	V600E	wt	wt		+/−	
	***Cell %/Localization***	40/Head-Neck	90/LN	80/LN			
**76**	***PNA***	V600	V600			+/+	
	***IHC***	moderate	strong			+/+	+/+
	***Capillary seq***	V600E	n.a.			n.a.	
	***Cell %/Localization***	70/Upper limb	90/LN				
**77**	***PNA***	WT	wt	wt	wt	−/−	
	***IHC***	negative	weak	weak	weak	-/+	−/−
	***Capillary seq***	WT	wt	wt	wt	−/−	
	***Cell %/Localization***	80/Upper limb	70/LN	60/LN	80/Mucosal		
**98**	***PNA***	wt	wt			−/−	
	***IHC***	negative	negative			−/−	−/−
	***Capillary seq***	wt	wt (k601k)			−/−	
	***Cell %/Localization***	80/Mucosal	90/Mucosal				
**100**	***PNA***	wt	wt			−/−	
	***IHC***	negative	negative			−/−	−/−
	***Capillary seq***	wt	wt			−/−	
	***Cell %/Localization***	n.a,/Trunk	n.a./LN				

Intrapatient overall concordance means that at least two techniques gave the same results in matched samples (M1, M2, M3: progressive matched metastatic samples; LN: lymph-node).

**Table 3:** Concordance between primary and metastatic samples as observed by three different methods and with the combination of at least two different techniques

	*BRAF* mutation				Concordance (%)
*Primary/Metastasis*	Concordant		Discordant		
	+/+	−/−	+/−	-/+	
***IHC***	8	5	1	1	87
PNA*	6	4	5	0	67
Sanger*	2	3	5	2	42
***At least two methods***	6	4	5	0	67

* when different metastatic samples were considered for each case, partial concordance was considered as discordance (n.a.: not applicable; +/+: both primary and metastatic samples *BRAF* positive; −/−: both primary and metastatic samples *BRAF* negative; +/−: primary sample *BRAF* positive and metastatic sample(s) *BRAF* negative; -/+: primary sample *BRAF* negative and metastatic sample(s) *BRAF* positive).

First, we considered the concordance of the results obtained with the three techniques, as we did for the primary samples: only 5/15 (33%) cases showed concordant results with all the three techniques; considering the results obtained combining the two most sensitive techniques, (IHC and PNA), we observed concordance in 67% of cases (10/15); if we considered at least two techniques we had concordant results in 100% (Table 2). Due to the small sample size (N < 30), inter-rater agreement for metastatic samples was not calculated in order to avoid false results.

Overall there was a high consistency between the data relative to *BRAF* mutation observed in primary and matched metastatic melanoma. Ten cases showed the same results between the primary and the metastatic sample, with the combination of the three methods (67% of concordance, considering a result obtained with at least two methods). The five discordant cases showed a *BRAF* mutation in the primary, which was not confirmed in the metastatic sample. The contrary was never observed (i.e. negative primary tumor showing *BRAF* mutation in the matched metastasis). Interestingly, 4 of the 5 discordant cases displayed metastatic lymph nodes which were not excised synchronously with the primary tumor.

When analyzing concordance per single method, we were able to verify that IHC showed results with the highest concordance (in 13/15 cases, 87%), followed by PNA (10/15 cases) with a concordance rate of 67% (the same observed with the combination of two methods). Instead, the concordance with capillary sequencing was very low (5/12, 42% of cases) (Table 3).

### NGS excludes that inconsistency between primary and metastatic sample is due to a low threshold of mutated alleles as detected in the primary tumor

NGS with a custom designed hot spot panel including *BRAF* gene was used to analyze 3 of the 5 primary/metastases discordant cases (n. 4, 13 and 25) in order to confirm our observations and to quantify the threshold of mutated alleles that could be identified through the other methods.

**Figure 2:**
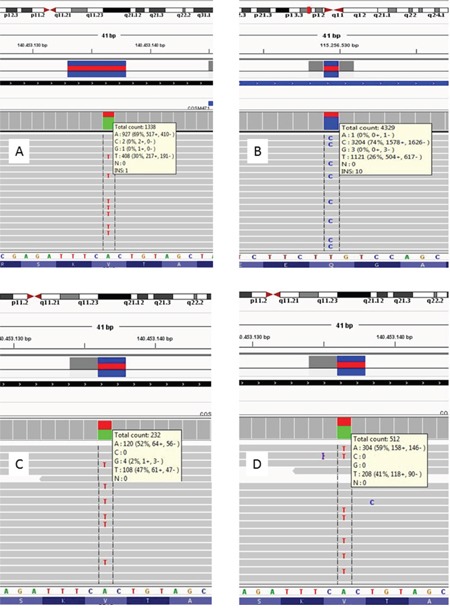
NGS results of 4 samples from three different patients **A.** BRAF NGS of Case 4 primary melanoma, which shows a mutant allele percentage of 30%. **B.** Case 4 melanoma metastasis, positive for NRAS Q61R mutation (mutant allele 74%); the same sample was BRAF wild type (not shown). **C-D.** BRAF results for other two primary melanomas (case 13 and 25), whose metastases were BRAF wild type; primary tissues presented 47% and 41% of BRAF mutant alleles respectively.

We confirmed the positive *BRAF* mutational status obtained with our previous results (Supplementary Table 1, Table 2), in primary melanoma at high rates (30, 47 and 41 % of mutated alleles, respectively) and lack of *BRAF* mutation in the metastatic samples (Figure 2A, 2C, 2D). Interestingly, in case 4 we observed a *BRAF* V600E mutation in the primary melanoma, undetectable in the paired metastasis, which instead revealed a *NRAS* Q61R mutation (Figure 2A, 2B). A possible explanation was that when we checked the history of this patient we found out that he was one of the 12 patients who developed multiple primary melanomas, before the development of metastatic disease (including the analyzed metastasis). Therefore, this non-consistency may be explained if the metastasis that we found did not originate from the same primary melanoma that we analyzed, but from the second primary melanoma (never tested because not available in our archives, but with a higher metastatic potential because of its Breslow level) that could have been NRAS-positive and *BRAF*-negative. For the other non-consistent pairs, no other primary melanomas were reported.

## DISCUSSION

Mutations in the *BRAF* oncogene are of increasing relevance in clinical oncology, especially after the approval of the anti-*BRAF* molecules in the therapy of *BRAF* positive metastatic melanomas. Therefore, finding the best technique to identify *BRAF* mutations with a high sensitivity and specificity is mandatory of accurate patient selection.

Here we tested BRAF mutations in a series of 100 primary melanomas with different techniques. Aim of our study was also to investigate intertumoral heterogeneity on the basis of the combination of different techniques used to detect *BRAF* mutations. Therefore, we analyzed 100 samples of primary melanomas using a combination of immunohistochemical analyses and real-time polymerase chain reaction (PNA in our cohort), in addition to classical capillary sequencing. IHC was more effective than capillary sequencing for detecting the V600E mutation. In a diagnostic setting it is the cheapest and the least time consuming technique (as it does not depend on DNA extraction) and can work on small melanoma samples.

CE-IVD PNA proved to be the most sensitive method in our cohort, as well as highly specific: it was also able to detect all mutations at codon 600, including the V600K mutation (as confirmed by capillary sequencing), while IHC antibody is marketed only for V600E identification. Due to its high sensitivity, PNA identified 67.3% of *BRAF* mutated samples, which is, to our knowledge, one of the highest percentages of *BRAF* mutations reported in the literature [4, 5, 7]. To our knowledge PNAs have been described one time only, very recently, for *BRAF* detection in melanomas; in addition they have been efficiently applied to *BRAF* testing for colorectal cancer and papillary thyroid carcinoma (PTC) [31, 37–40].

Two issues may justify this high percentage of mutated samples: the high sensitivity of PNA (compared to IHC and most to Sanger Sequencing) and the high prevalence, in our population, of melanomas in sites not chronically sun exposed (CSE) as trunk and limbs (Table 1), which are known to be associated with *BRAF* mutation [41].

The use of three different methods was useful in identifying the method with higher sensitivity and concordance compared to the others. Data obtained with IHC and PNA were quite consistent, while data obtained with capillary sequencing confirmed the difficulty in identifying a mutated allele when the percentage of tumor cells was below a threshold (which is generally considered to be 50%), in addition to the difficulty of working with little DNA, as in small primary melanoma, limiting PCR amplification prior to sequencing.

We conclude that PNA testing in primary melanoma is highly specific and reliable in detecting even a small fraction of mutated allele, undetectable with other methods (Figure 1F) making PNA, which is CE-IVD approved, applicable in clinical practice. Limitations in the choice of this technique could be its higher cost if compared to IHC [31] and dependence on DNA extraction.

While *BRAF* V600E mutation appears to play a critical role in tumor initiation, its expression during tumor progression remains controversial. Various authors claim that *BRAF* V600E heterogeneity present in melanoma, could be due to tumor sampling, the detection method used, or secondary to biological differences [18, 21, 22, 24, 43, 44]. Since coexistence of *BRAF* wild-type and *BRAF* mutant tumor cells within the same patient has important implications for clinical decisions, more sophisticated tools are needed to characterize patients’ cancers and guide their treatment.

With the aim to evaluate the issue of possible intertumoral heterogeneity in our cohort, we used the same three-methods approach, mentioned above, on 25 matched primary and metastatic samples from 15 patients in our cohort. In several papers, relying mainly on IHC, *BRAF* expression is assumed to be homogeneous in the majority of the lesions from the same patients [17, 23]. In our cohort, a consistency of nearly 90% was found using IHC, which supports this observation (Table 3). The addition of molecular techniques further clarifies that the observed heterogeneity is not due to the single techniques used. We observed that 10 (67%) cases showed consistency between primary and metastases for *BRAF* mutational status (6 mutated and 4 wild type), when assessed with at least two methods. In 5 cases there was no consistency, all the cases had a *BRAF* mutation in the primary which was not identified in the subsequent metastases. This result partially supports recent large studies in which, in the majority of cases, the primary and metastatic lesions had the same *BRAF* genotype [20, 45]. Indeed, we observed a good but not complete consistency between primary and metastatic samples from the same patients (10/15) while different metastatic lesions from the same patients revealed a concordant *BRAF* status.

The finding that *BRAF* mutations in primary melanomas is not always identified in the correspondent metastases can also partly explain the high percentage of *BRAF* mutations observed in our cohort of primary tumors (62% with at least two methods) compared to the literature, in which only metastatic tissue is evaluated. In addition, the natural history of the disease and the occurrence of multiple primary melanoma in the same patients should always be considered.

Overall, despite our 15 patients analyzed represent the 68% of the cases which relapsed, our results are limited by the small numbers and the mutation testing of only for *BRAF* gene.

While it is still unclear what is the selective pressure that induces migration of a *BRAF*-wild-type subclone instead of an expected more aggressive *BRAF*-mutant subclone, an approach based on NGS targeting multigene panels could help in clarifying this issue. Finally a recent study using NGS identifies very low heterogeneity in matched primary and lymph node metastatic samples, especially in synchronous metastases, while heterogeneity is more frequent in metachronous lesions [46]. Our results, using the concordance between at least two methods, confirm this finding, since our discordant cases displayed metastatic lymph-nodes which were not excised synchronously with the primary tumor.

In conclusion, the intertumoral (primary and metastatic) heterogeneity described here is not attributable to technical issues when molecular methods are used, since it was observed with a combination of different technique and partially confirmed by a fourth, quantitative, technique as NGS.

A combination of IHC, used as a screening tool, and parallel formal molecular mutation testing seems to provide the highest reliability for identifying the majority of *BRAF* mutated cases with the highest sensitivity and specificity [47, 48]. The use of IHC alone, without parallel molecular testing should be limited to samples of small size or with low tumor cell content for optimal DNA extraction. Conversely, the use of very sensitive molecular techniques, which could detect a minor *BRAF*-mutated subclone in a predominantly wild-type tumor, may not be clinically relevant as BRAF inhibitors may have opposite signaling effects in cells with mutated or wild-type *BRAF* [23].

The limits of this approach are in the comparison of techniques with very different sensitivity (IHC vs PNA) and specificity with the capability to identify different cellular structure (proteins for IHC; DNA for molecular techniques). In addition, when a second molecular technique is necessary, the increase in turnaround time and costs is another possible limit to this type of approach.

**Figure 3:**
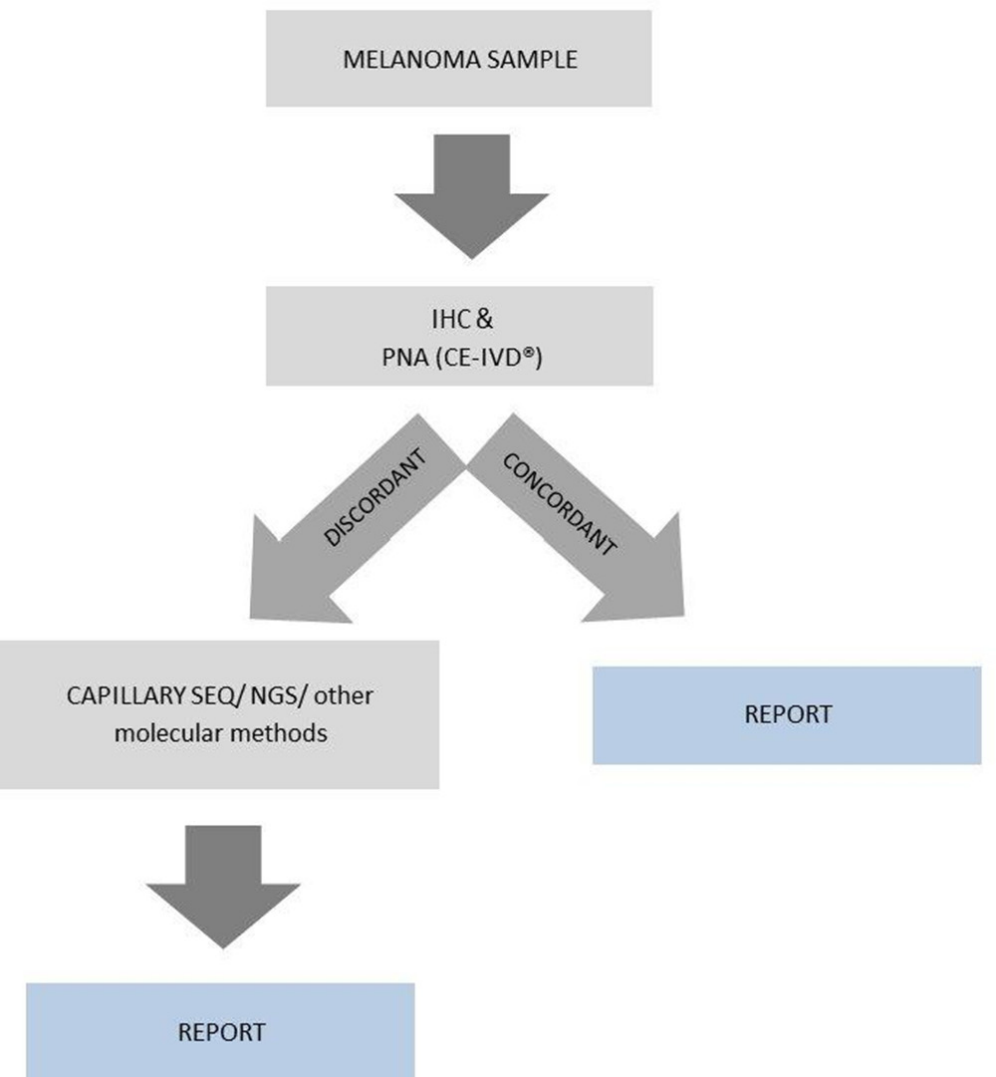
Diagnostic algorithm proposed for BRAF testing in metastatic melanoma Report is based on the results obtained with two concordant methods. Applicable also to primary tumors when needed.

In clinical practice we propose the analysis of the most recent and clinically relevant sample (metastatic when available) with IHC and at least one molecular method (in our case we propose PNA). If *BRAF* mutation is identified in PNA, but not with IHC a third analysis with another method (in our case capillary sequencing or, for a quantification, NGS or pyrosequencing) is recommended in order to obtain a concordance of at least two methods, or identification of another *BRAF* V600 mutation which is undetectable with VE1 antibody (Figure 3). Testing the primary tumor when the metastatic sample is unavailable is a good option if at least two methods of detection are used, however the presence of intertumoral heterogeneity or the occurrence of additional primaries should be carefully considered.

Interestingly, a recent study suggests that the staining pattern (homogeneous vs. heterogeneous) might be associated with differences in therapeutic response to BRAF inhibitors [19]. This is a field which will need further investigation in the near future and parallel IHC testing, which is currently being developed with antibodies directed to other *BRAF* or *NRAS* mutations, is an advantageous opportunity.

## MATERIALS AND METHODS

### Case selection

A total of 137 melanoma samples were included. 100 primary melanomas were selected based on formalin-fixed, paraffin embedded (FFPE) tissue availability at the Pathology department, IRCCS AOU San Martino IST, Genoa.

All melanoma patients had been referred to our center for germline testing either for diagnostic (familial and multiple melanoma cases) or research purposes (sporadic cases from an ongoing case-control study [26–28]). All the patients signed an informed consent under local IRB approved protocols for both germline testing and other research purposes dealing with the archived melanoma tissues in the pathology department. A local database collecting information from the pathology report, tumor cell content, in the examined section, and follow-up was designed. The database is regularly updated with the oncologists’ and local registries database, and includes detailed information on relapse, survival, time and cause of death.

A total of 25 metastatic samples, from 15 patients, were available and tested with the same techniques used for the primary tumors. Twelve metastatic samples, already tested for diagnostic purposes by molecular techniques, were used as positive and negative controls for IHC assessment (4 *BRAF* positive e 8 negative).

### DNA extraction

All diagnoses were performed by two expert dermatopathologists (FC and MG), and subsequently reviewed for tumor content, neoplastic cell morphology (epithelioid vs spindled) and immunohistochemistry evaluation (LM and FG).

The tumor area was identified, and marked by the pathologists, on hematoxylin and eosin stained slides. Marked tumor tissue was manually dissected from two to six slides (8-micron thick) of FFPE tissue for each case.

Genomic DNA was extracted from the sections using the QIAmp DNA FFPE tissue kit (Qiagen, Valencia, CA, USA) or the Genomic DNA FFPE One-Step Kit for Diatech MagCore® HF16Plus extractor (RBC Bioscience, New Taipei City, Taiwan), in both cases according to the manufacturer's instructions. Quality and concentration of the DNA samples were examined by SPECTROstar Nano (BMG Labtech, Offenburg, Germany).

### Capillary sequencing

Capillary sequencing was performed as previously described [29–31]. Briefly, we amplified tumor DNA in a 15 μl PCR containing approximately 40 ng of purified genomic DNA in a mixture of 1.5 μl of Buffer (10x), 1.5 μl dNTP (2 mM), 0.08 μl Qiagen HotStart Taq (5U/μl) and 2 μl forward and reverse primer (5 mM). After denaturation at 95°C for 15 minutes, 35 amplification cycles at 95°C for 40s, 60°C for 30s, 72°C for 50s followed by elongation at 72°C for 10 minutes were performed.

Sequencing was conducted with Big Dye Terminator sequencing kit v1.1 (Life Technologies, Carlsbad, CA, USA) according to the manufacturer's instructions, and sequencing reactions were electrophoresed on an ABI 3130xl genetic analyzer (Applied Biosystems, Carlsbad, CA, USA). Sequencing reactions were repeated at least twice by independent PCR, with forward and reverse primers, and the sample was scored as being mutated when the mutation was observed both times. *BRAF* primers for amplification of exon 15, used for both PCR and Capillary sequencing were previously described [31].

### PNA clamping quantitative PCR analysis

*BRAF* V600 codon mutational status was tested using the PNAClamp™ *BRAF* Mutation Detection Kit (Panagene, Daejeon, Korea), a CE-IVD certified technique based on peptide nucleic acid (PNA)-mediated real-time PCR clamping technology. PNA is a synthetic DNA analog in which the phosphodiester backbone is replaced by a peptide-like repeat formed by (2-aminoethyl)-glycine units. The technique is based on the principle that PNA inhibits wild type by hybridizing normal sequences, and therefore mutant DNA is preferentially amplified.

The reaction was performed according to the manufacturer's instructions, with slight modifications, as previously described [31]. The threshold cycle (Ct) was automatically calculated from the PCR amplification plots where fluorescence was plotted against the number of cycles. Delta-Ct values were calculated as the Ct values of the samples minus those of the controls. The higher delta-Ct values showed that the mutant was efficiently amplified. A cut-off value of 2.0 was used to determine the presence of mutant DNA.

### Immunohistochemistry

Immunohistochemistry was performed using the *BRAF* V600E mutation-specific antibody (Springer-Bio, clone VE1, 1:50 dilution). Four micron-thick tissue sections were freshly cut [32], dried, deparaffinised and rehydrated. Endogenous peroxidase was blocked with 5% H_2_O_2_ for 10 minutes. Immunoreactions were performed using the automated BenchMark XT immunostainer® (Ventana Medical Systems, Arizona, USA). Standard heat-based antigen retrieval was performed for 30 minutes. The ultraVIEW Universal Alkaline Phosphatase Red Detection Kit (Ventana Medical Systems, Arizona, USA), was used. After immunostaining, slides were counterstained with haematoxylin and coverslipped. All reactions were carried out on the same day on consecutive runs and positive and negative controls were added for each run. Positive and negative controls used were chosen from metastatic samples already characterized for *BRAF* molecular status for diagnostic purposes (both Capillary sequencing and PNA clamping real-time PCR, as performed by the manufacturer).

All immunostained slides were simultaneously scored by two pathologists (LM and FG) blinded to the *BRAF* status; disagreement was resolved by consensus. IHC was considered positive when BRAF V600E cytoplasmic protein expression was scored according to the 3 categories score used by Tetzlaff et al [33]. Briefly, negative staining was defined as either absence of any cytoplasmic labeling or nuclear staining only in melanoma cells; positive staining was distinguished in either positive homogeneous (staining in > 95% of cells) or positive heterogeneous (staining in < 95% of cells) based on percentage of cytoplasmic staining in melanoma cells; intensity of staining was also scored as weak, moderate or strong.

### Next generation sequencing

Re-analysis of primary and metastatic samples showing discordant results with the other techniques from 3 selected patients was performed by targeted next generation sequencing (NGS) as previously described [31]. Briefly, 10 nanograms of DNA were amplified with an in-house, customized Ion AmpliSeq primer tool, comprising 32 amplicons of 19 genes, targeting 79 hotspot mutations, including the hotspot exon 15 of the *BRAF* gene. PCR products were ligated to adapters and enriched for target regions using the Ion AmpliSeq™ Library Kit 2.0 according to the manufacturer's instructions (Thermo Fisher Scientific, Waltham, Massachusetts, USA). The samples were quality checked for proper amplicon length and quantity on the 2100 Bioanalyzer Instruments (Agilent Technologies, Santa Clara, California, USA), then sequenced on the Ion Personal Genome Machine™ (PGM™) System. Data were analyzed using the Torrent Suite Software, version 5.0.4.0 (Thermo Fisher Scientific, Waltham, Massachusetts, USA) and visualized in the integrative genome viewer (IGV); annotation was performed by Ion Reporter™ Software (Thermo Fisher Scientific, Waltham, Massachusetts, USA).

### Inter-rater agreement

To measure agreement between 3 techniques (Sanger sequencing, PNA and IHC) in determining BRAF mutational status, we computed the Light's Kappa coefficient [34]. The analysis was performed using the IRR package [35] within the R computational environment (R Foundation for Statistical Computing, Vienna, Austria) [36].

## SUPPLEMENTARY MATERIALS FIGURES AND TABLES



## Abbreviations

PNA: peptide nucleic acid –PNA-clamping real-time PCR
IHC: immunohistochemistry
NGS: next generation sequencing
FFPE: formalin-fixed, paraffin embedded
