# Supplementary material for: Heterogeneity and frequency of BRAF mutations in primary melanoma: Comparison between molecular methods and immunohistochemistry
Source: Oncotarget. 2016 Dec 21;8(5):8069–82. doi: 10.18632/oncotarget.14094 (PMC5352383; doi:10.18632/oncotarget.14094)
Supplement: Supplementary file 1 [file oncotarget-08-8069-s001.pdf]

Heterogeneity and frequency of *BRAF* mutations in primary melanoma: Comparison between molecular methods and immunohistochemistry

SUPPLEMENTARY TABLE

Supplementary Table 1: Neoplastic cells content and BRAF mutational status (assessed with PNA-clamping real-time PCR, capillary sequencing and immunohistochemistry) in our 100 primary melanomas cohort

See Supplementary File 1
